# Effect of Melatonin on Cardiac Injury after Primary Percutaneous Coronary Intervention: a Randomized Controlled Trial

**Published:** 2015

**Authors:** Padideh Ghaeli, Shaghayegh Vejdani, Atefeh Ariamanesh, Azita Hajhossein Talasaz

**Affiliations:** a *Department of Clinical Pharmacy, Faculty of Pharmacy, Tehran University of Medical Sciences, Tehran, Iran.*; b *Roozbeh Hospital, Tehran University of Medical Sciences, Tehran, Iran.*; c *Tehran Heart Center, Tehran University of Medical Sciences, Tehran, Iran.*

**Keywords:** Melatonin, Myocardial infarction, Primary percutaneous coronary intervention, High sensitive Troponin-T, Creatine phosphokinase-MB

## Abstract

Several studies have reported that the antioxidant properties of melatonin can provide cardiac protection through scavenging of free radicals. This study sought to investigate the efficacy of melatonin on cardiac biomarkers, myocardial-specific protein high sensitive troponin-T (hs-TnT) and creatine kinase-MB (CK-MB), in patients with ST-segment elevation myocardial infarction (STEMI) undergoing primary percutaneous coronary intervention (pPCI). In this randomized clinical trial, a total of 40 patients with STEMI planned to undergo pPCI were randomly assigned to two groups of receiving melatonin plus standard treatment [n=20] and control group, receiving only standard therapy [n=20]. The following parameters including hsTnT and CK-MB were assessed preoperatively (baseline) and at 6 hours after procedure. Melatonin could significantly reduce the level of CK-MB (118.2 ± 21.09 IU/L in the treated group versus 198.24 ± 20.94 IU/L in the control group; p-value = 0.01). However, there was no difference in the mean hs-TnT level between two groups (2491 ± 664 μg/L vs. 2801 ± 620 μg/L; p value = 0.73). Our results revealed that melatonin can be considered as a safe adjunctive medication to the standard regimen after pPCI for the aim of decreasing cardiovascular events. Meanwhile, this was a pilot study with a small number of patients and further studies are needed to confirm the beneficial effect of melatonin in patients with STEMI.

## Introduction

Early reperfusion with thrombolytic therapy or primary percutaneous coronary intervention (pPCI) is the most effective strategy for the patients with an acute ST-segment elevation myocardial infarction (STEMI). In contrast, the process of reperfusion leads to injury which can induce myocyte death. During the period of reperfusion oxygen-derived free radicals, both reactive oxygen species (ROS) and reactive nitrogen species (RNS) are produced that lead much of myocardial damages. They alter calcium signaling in platelets, and stimulate aggregation (1, 2).

Antioxidants such as allopurinol, vitamin E and pentoxiphylline could have myocardial protection via scavenging of free radicals (3). Because of melatonin’s potent antioxidative and anti-inﬂammatory properties, it can exhibit significant effect in scavenging ROS/RNS-induced cellular toxicity. Also, Melatonin stimulates antioxidant enzymes including glutathione peroxidase and superoxide dismutases (cytosolic and mitochondrial) (4).

A study by Zaslavskaia RM, *et al*. compared 3 groups of patients suffering arterial hypertension (AH) and coronary heart disease (CHD). Patients in one group were receiving melatonin, subjects in the second group were receiving combined treatment (CT) of melatonin with antihypertensives and patients in third group were only on antihypertensives. The clinical examinations and evaluations of oxidative and antioxidative activities in erythrocytes were performed before the initiation of the study and 21 days after. Treatment efficiency was compared between the groups. The results of this study suggested that efficiency of using antihypertensives alone was inferior to that of melatonin or CT in these patients. Patients in melatonin and CT groups showed more marked anti-ischemic and anti-angina effects as well as more normalized oxidant/antioxidant balance (5).

High sensitive Troponin T (hs-TnT) and the creatine kinase-MB (CK-MB) are the most sensitive biomarkers for detecting myocardial cell damage during pPCI (6, 7). 

Therefore, this preliminary study was designed to investigate the effects of short-term use of Melatonin on cardiac biomarkers, troponin-T and CK-MB in patients with STEMI undergoing pPCI.

## Experimental

*Material and methods*

The study protocol was approved by the Ethics Committee of the Tehran University of Medical sciences, and informed consent was obtained from all patients. Forty patients, between July 2012 and April 2013, with STEMI underwent pPCI accepted to enter this randomized clinical trial.

Patients who received medications that could affect sleep pattern such as benzodiazepines, tricyclic antidepressants, antipsychotics, xanthenes, dopamine agonists and  -agonists were excluded from this study.

The method of randomization was permuted random block. Total patients were randomly assigned into two groups: those receiving melatonin 3 mg (Naturemade, USA) plus standard treatment [test] and those who receive only standard therapy [control]. Melatonin was administered through the period of hospitalizations for each patients. The standard drug therapy in our heart center contains Nitrates (TNG infusion), Aspirin 300mg then 80 mg, Clopidogrel 600 mg then 75 mg, Beta blockers (Metoprolol dosed based on patient’s hemodynamics), Angiotensin converting enzyme (ACE) inhibitors (Captopril 6.25 mg three times daily which is titrated based on patient’s response), Statin (Atorvastatin 40 mg twice daily), and Heparin 4000 IU stat followed by 1000 IU per hour.

 hs-TnT and CK-MB were measured at the following time points: preoperatively (baseline) and at 6 hours after procedure.

SPSS software version 16 was used to analyze the data. T-test was performed to compare the differences in mean values of troponin T and CPK-MB between the 2 groups at baseline and 6 hours later. Results are presented as mean ± standard deviation (SD). The p-value of less than 0.05 was considered statistically significant.

## Results

Patients were randomized in two groups of 20 each: those who received melatonin (test) and those who do not received melatonin (control). The range of age of our patients was 38 to 80 years in the control group and 40 to 83 years in the test group. The male/female ratio was 15/5 in the control group and 15/5 in the test group. Baseline clinical demographic proﬁles of patients are shown in Table 1. As shown in this table, the differences between test and control groups were not statistically significant.

**Table 1:** The comparison between baseline characteristics of the patients in each group

Baseline parameters	**Melatonin group**	**Control group**	**p-value**
Age (years)(Mean±SD)	58.64 ± 12.91	58.13 ± 11.87	0.94
Sex			
Male	15	15	1.000
Risk Factors			
Smoking	6	7	0.74
Hypertension	15	13	0.50
Dyslipidemia	8	7	0.75
Diabetes	6	7	0.74
Family History	7	8	0.75
Pervious MI	2	1	0.56
Pervious PCI	1	1	1.000
Drug History			
ACEI	11	9	0.54
ARB	3	4	0.68
Statins	8	7	0.75
Aldacton	1	0	0.32
Beta-blockers	8	6	0.52
Asprin	17	16	0.63
Clopidogrel	3	2	0.64
Pre-hospital delay(min)	177.50 ± 58.57	174.5 ± 55.86	0.86

ACEI: Angiotensin converting enzyme inhibitor; ARB: Angiotensin receptor blockedr; MI: Myocardial Infarction, PCI: Percutaneous Coronary Intervention Hypertension was defined in this study as BP≥ 140/90 mmHg, Dyslipidemia defined as either elevated total, LDL cholesterol, TG levels, or low levels of HDL cholesterol, Diabetes was defined as two FBS levels of upper than 126 mg/100cc, Positive family history means the presence of premature CHD in first degree male relatives below the age of 55 years or female relatives below the age of 65 years.

*The difference between biomarkers*

The mean± SD of hs-TnT level was 2801 ± 620 μg/L in the control group and 2491 ± 664 μg/L in test group, without a significant statistical difference (p-value=0.73). 

No significant difference was noted in the baseline CKMB between the two groups of the present study (CKMB of the control group was 23, 55 ± 25, 11 IU/L versus 22.28 ± 33.84 IU/L for the treatment group; p value = 0.89). The mean±SD CK-MB levels were significantly different between control and test groups (198.24 ± 20.94 IU/L in the treated group versus 118.2 ± 21.09 IU/L in the control group; p-value = 0.01)

## Discussion

Infarct size and the percent of necrosis are important factors affecting survival after acute myocardial infarction (AMI) (2, 8). Primary PCI is an appropriate strategy of reperfusion for patients with STEMI when it can be performed as soon as possible (9). Despite this, one of the negative aspects of myocardial reperfusion process is myocardial reperfusion injury. Several pathophysiological mechanisms, usually in combination, are responsible for this irreversible event such as generation of oxygen by products and platelets aggregation (1, 10). 

It is believed that antioxidant as a cardioprotective therapy can reduce reperfusion injury in STEMI patients. One of the vital factors of usefulness of antioxidants is that they must have the ability of penetrating the cell membrane and to scavenge free radicals in situ. Melatonin can diffuse through the cell membrane easily in contrast to other antioxidants, like vitamin E or vitamin C. Also, melatonin is two times more effective at scavenging the peroxyl radical than vitamin E (11).

Melatonin is a strong scavenger of the free radicals and could limit myocardial infarct (MI) size and necrosis after cardiovascular procedures. Moreover, metabolites of melatonin such as *N*-acetyl-5-methoxykynuramine and *N*1-acetyl-*N*2-formyl-5-methoxykynuramine are active metabolites that can scavenge free radicals. They not only induce production of antioxidant enzymes but also prevent generation of pro-inflammatory and pro-oxidative enzymes (12). 

The level of melatonin decreases with age, age-related diseases and in many other diseases that involves reactive oxygen and/or nitrogen species as their etiology including cardiovascular disease (13).

Data from several animal studies suggest that melatonin can provide the myocardial protection against ischemic/reperfusion injury (14). 

In a cohort study, serum levels of melatonin and oxidative stress parameters were measured in 25 patients with acute myocardial infarction and 25 controls without coronary artery disease. This study reported that there is an association between acute myocardial infarction and the nocturnal serum melatonin deficit (15). In our study we observed that patients received melatonin had lower oxidative stress parameters, so melatonin administration resulted in less myocardial infract size in comparison to control group.

In another study, a correlation between melatonin concentration and ischemia-modified albumin in STEMI patients underwent pPCI were studied. Ischemia-modified albumin was used as a marker of myocardial ischemia. The results suggested that melatonin can limit the cardiac damage induced by ischemia-reperfusion through its antioxidant properties (16). Our results werein line with this study, as the cardiac biomarkers were more balanced in a group who received melatonin.

In a prospective trial, the hypothesis tested that whether melatonin can eventually increase myocardial salvage and improve clinical outcome when given as an adjunct to reperfusion treatment in patients with acute myocardial infarction. They asserted that melatonin can confer cardioprotection against ischemia-reperfusion injury. So, if the study will be successful, the finding would support the use of melatonin in therapy of ischemic-reperfusion injury of the heart (17). The difference between the design of this study and ours is that in our study patients received the melatonin 3 mg from the first night after the P-PCI orally. It is obvious that giving melatonin before P-PCI procedure could have more significant effects on myocardial cells.

A relationship between the ‘‘no-reflow’’ phenomenon and intraplatelet melatonin content in patients with STEMI after pPCI were assessed in a clinical trial. Lower levels of intraplatelet melatonin were observed in patients with angiographic no-reflow. Also, these patients have higher systemic oxidative stress (18). We didn’t measure the level of melatonin. However, the data of our study suggested that melatonin can limit the myocardial damage induced by ischemia-reperfusion through its antioxidant properties.

In this study we investigated the effect of Melatonin on cardiac injury in patients with STEMI undergoing primary PCI. Our results show that patients receiving Melatonin have better condition as compared to their counterparts who did not receive melatonin.

Our study was not without limitations. The duration of this study, the number of participants and small doses of melatonin compared with some other studies can be the limiting factors.

## Conclusion

This study revealed that melatonin may be able to reduce the incidence of adverse cardiac events by cardiac protection through scavenging of free radicals. However, further studies with large sample size are needed to confirm the beneficial effect of melatonin in patients with STEMI.
